# Tetanus Treated by Woorali, Calabar Bean, and Chloral Hydrate

**Published:** 1871-02

**Authors:** 


### Tetanus treated by Woorali, Calabar Bean, and Chloral Hydrate.

Mr. Lawson Tait records (Lancet, Oct. 1, 1870,) three eases of traumatic tetanus; the first treated by woorali, the second by Calabar bean, and the third by chloral hydrate. He very justly remarks that "perhaps there is no disease about which men rush more ardently into print than tetanus; a single case often constitutes a paper, and from it other practitioners are led to use the vaunted remedy, only to meet with disappointment, and few record it. It has been my misfortune to see a good deal of tetanus, and I have tried many remedies, but always with the same result. If the patient lives over the twelfth day, he is almost certain to recover, whatever be the treatment. The acute cases terminate in from twenty-four hours to three days, and nothing seems to help them in the least, except, perhaps, that chloral h drate gives them an easier death than they have without it. The last three cases of acute tetanus which I have seen I have treated severally by woorali, Calabar bean and chloral hydrate. The results have been unsatisfactory, as usual, and in the first case, treated by woorali, I believe that death was, if an ything, hastened by the treatment. In these cases, elaborate notes were taken of the temperature, pulse, etc., but as they reveal nothing not already well known, they are omitted."

"In these cases," he adds, "it cannot be said that the treatment was of the slightest use, except that in the last case the chloral saved much suffering, both to the patient and his friends. This could be formerly done with chloroform, but with much more trouble, and with no more satisfactory result. I fear that even chloral is not to prove of the value in the treatment of this terrible disease which some of the members of the French Academy recently predicted for it. The other two drugs are still more useless."

